# Risk of Microvascular Complications and Macrovascular Risk Factors in Early-Onset Type 1 Diabetes after at Least 10 Years Duration: An Analysis of Three Population-Based Cross-Sectional Surveys in Germany between 2009 and 2016

**DOI:** 10.1155/2018/7806980

**Published:** 2018-04-01

**Authors:** Thaddäus Tönnies, Anna Stahl-Pehe, Christina Baechle, Katty Castillo, Oliver Kuss, Rhuphine Yossa, Lena M. E. Lindner, Reinhard W. Holl, Joachim Rosenbauer

**Affiliations:** ^1^Institute for Biometrics and Epidemiology, German Diabetes Centre (DDZ), Leibniz Centre for Diabetes Research, Heinrich Heine University, Auf'm Hennekamp 65, 40225 Duesseldorf, Germany; ^2^German Centre for Diabetes Research (DZD), Ingolstädter Landstraße 1, 85764 Neuherberg, Germany; ^3^Institute of Epidemiology and Medical Biometry, University of Ulm, Albert-Einstein-Allee 41, 89069 Ulm, Germany

## Abstract

**Aims:**

To estimate the risk of microvascular complications and macrovascular risk factors among persons with early-onset (diagnosed at ages 0 to <5 years) and long-duration type 1 diabetes and determine temporal trends and associations with potential predictors.

**Methods:**

We conducted three population-based cross-sectional surveys in Germany (*N* = 1789) to obtain information on exposures and five outcomes (retinopathy, nephropathy, dyslipidemia, hypertension, and a composite endpoint combining all four outcomes). For each outcome, log-binomial spline regression was applied to estimate the risk and dose-response relationship with diabetes duration and exposures.

**Results:**

The risk for microvascular complications increased after 14 years since diabetes diagnosis whereas dyslipidemia and hypertension were already prevalent at 10 years. The 15-year risk (95% confidence interval) of the composite endpoint for female and male patients was 22.9% (18.8%–27.9%) and 19.2% (15.5%–23.8%), respectively. Temporal trends suggested a decreasing risk between 2009 and 2016. Glycemic control, lifestyle-related factors, and SES, but not health care-related factors, were associated with the risk of the composite endpoint.

**Conclusions:**

In early-onset type 1 diabetes, there exists a considerable risk of complications and comorbidities already in young ages. Future research should focus on prevention of diabetic complications in young patients and clarification of pathways of the associations found.

## 1. Introduction

During the past decades, many advances in routine therapy of type 1 diabetes have been achieved, for example, use of insulin pumps, glucose sensors, insulin analogues, or intensified diabetes education and psychosocial support [1]. Evidence suggests that the risk decreased for some, but not all, diabetes-related complications [2, 3]. For example, microvascular complications such as diabetic retinopathy (DR) and diabetic nephropathy (DN) still play an important role in the clinical course of type 1 diabetes. DR with pathologic changes of retinal vessels is the most frequent microvascular complication and may lead to blindness in advanced stages [4]. According to Kaplan–Meier analysis, the cumulative proportion of any DR after 40 years of diabetes duration was estimated at 84% [5]. DN with glomerular vascular alterations is a major cause of end-stage renal disease requiring dialysis or kidney transplantation [4, 6, 7]. DN is characterized by progressive stages of proteinuria with microalbuminuria as the mildest form [4]. The crude risk of micro- and macroalbuminuria or end-stage renal disease is estimated with almost 25% and 9%, respectively, after 40 years of diabetes duration [8].

Besides microvascular complications, type 1 diabetes is still associated with an increased risk of cardiovascular disease (CVD) and associated mortality [9]. Accordingly, the prevalence of CVD risk factors is still high among patients with type 1 diabetes. For instance, Schwab et al. [10] found that 69% of persons with type 1 diabetes aged 0 to 26 years had at least one risk factor for CVD. Further, the number of risk factors increased with age.

Since the DCCT trial, the central aim of near-normal glycemic control is well established to avoid diabetes-related late complications and comorbidity [11]. Besides that, social, lifestyle-related, and health care-related factors have been associated with the risk of complications and/or CVD risk factors [12]. In turn, glycemic control has been related to psychosocial and family background in children and adolescents [12, 13].

In this study, we focus on the risk of diabetic microvascular complications and macrovascular risk factors after the onset of type 1 diabetes in preschool age and at least 10 years of diabetes duration. This patient group needs special focus since the incidence of type 1 diabetes in this age group has been predicted to further increase in many European regions [14]. Due to the early onset of type 1 diabetes, the increased risk of micro- and macrovascular diseases may occur early in life imposing a potentially large number of life years lost and years lived with disability. Additionally, the challenges of puberty may hamper self-management of type 1 diabetes, which may affect patterns of risk factors for complications. Therefore, this analysis aimed to (i) estimate the risk of microvascular complications (diabetic retinopathy, nephropathy) and macrovascular risk factors (hypertension, dyslipidemia) among persons with early-onset type 1 diabetes, (ii) determine temporal trends of these risks, and (iii) quantify the association between the risk and health care-related factors, socioeconomic status (SES), glycemic control, and lifestyle-related factors.

## 2. Research Design and Methods

### 2.1. Study Design and Data Source

We used three population-based cross-sectional baseline surveys (2009/2010, 2012/2013, and 2015/2016) of the German cohort study “Clinical Course of Type 1 Diabetes in Children, Adolescents and Young Adults with Disease Onset in Preschool Age” (type 1 diabetes study). Potential study participants with type 1 diabetes onset prior to the age of 5 years and with at least ten years diabetes duration were identified from the nationwide early-onset type 1 diabetes registry at the German Diabetes Center, Düsseldorf (Deutsches Diabetes-Zentrum, DDZ). The completeness of the registry is estimated to be 97% [15]. Standardized self-administered age-adapted questionnaires were sent to eligible type 1 diabetes patients via treating facilities having formerly reported cases to the type 1 diabetes registry. In case of participants being under 18 years of age, parents also received a questionnaire. Nonresponders were asked to fill out a short questionnaire. Further information on the type 1 diabetes study has previously been reported by Stahl et al. [16]. The studies were fully approved by the ethics committee of Düsseldorf University (reference number 3254).

### 2.2. Variables

#### 2.2.1. Outcome Variables

Outcomes investigated were DR, DN, hypertension, and dyslipidemia. An outcome was considered present if the participant or the participant's parents reported that the respective outcome had ever been diagnosed by a physician. If the outcome was reported to have never been diagnosed, the outcome was considered nonpresent. To increase the statistical power, we also considered a composite endpoint evaluated as present if the participant or the participant's parents reported that at least one of the four outcomes had ever been diagnosed by a physician. The composite endpoint was evaluated as nonpresent if none of the four outcomes had ever been diagnosed by a physician.

#### 2.2.2. Exposure Variables

Besides time trends, we investigated SES, family structure, lifestyle-related variables (body mass index (BMI), physical activity (PA)), glycemic control (HbA1c, self-monitoring of blood glucose (SMBG), and number of omitted insulin injections (OII)), and health care-related variables (continuous subcutaneous insulin injection (CSII), participation in a disease management program (DMP), and use of diabetes health card (DHC)) as exposure variables.

Time trends were investigated by comparing the prevalence of the outcomes in the three surveys (2009/2010, 2012/2013, and 2015/2016). Diabetes duration was defined as the time between type 1 diabetes diagnosis and the completion of the questionnaire and evaluated as a continuous variable. Due to the small range of onset age (0–<5 years) in our cohort, age is highly correlated with diabetes duration. Therefore, we only included diabetes duration in the analyses assuming that diabetes duration probably has a greater impact on the risk of complications than age.

We measured SES on the household level using the Winkler index, which has also been used in the German child and adolescent health-monitoring study [17]. The index combines scales for income, education, and occupation into a continuous score ranging from 3 (lowest SES) to 21 (highest SES). Family structure was defined as a dichotomous variable and distinguished between participants living with both biological parents versus all other constellations (e.g., living alone/in own apartment, with foster parents, and in a children's home).

BMI was calculated as body weight in kilograms divided by squared height in meters (kg/m^2^). To account for the variability of the BMI in young ages, we calculated BMI standard deviation scores (BMI-SDS). BMI-SDS was derived based on reference data from the German Working Group Adiposity using the Lambda-Mu-Sigma method [18, 19]. In the analyses, BMI-SDS was included as a continuous variable. PA was defined according to the question “How often are you physically active in your leisure time such that you really get to sweat or get out of breath?” We distinguished the four ordinal categories never, 1-2 times/month, 1-2 times/week, and more than twice a week.

We used three measures to assess glycemic control and self-management of glycemic control. First, the self-reported and most recently measured glycated hemoglobin (HbA1c) value in percent of total hemoglobin was evaluated as a continuous variable. In cases of different HbA1c values being reported by participants and parents, we calculated the mean of both values. SMBG referred to self-reported average daily frequency SMBG during the last three months. In the analyses, SMBG was included with four categories (0–2, 3–5, 6–8, and >8 measurements/day). The frequency of OII during the last week was used as an indicator for treatment adherence. OII was based on the self-reported frequency of insufficient or omitted insulin injections at an occasion of carbohydrate consumption during the last week.

Differences in health care were measured by three indicators. First, we distinguished participants with regard to their insulin therapy regimen using the three categories: 1–3 injections/day, at least 4 injections/day, and CSII. Second, we distinguished whether or not participants took part in a DMP, as structured model of diabetes care. In Germany, DMPs are provided by health care providers in cooperation with health insurances [20]. Third, we distinguished whether or not participants used the DHC. The DHC aims to support the monitoring of critical parameters regarding process and outcome quality in order to avoid late sequelae of diabetes. German national guidelines recommend the DHC as part of structured educational programs for persons with type 1 diabetes [21].

### 2.3. Statistical Analysis

Participants with missing values for all outcome variables were excluded from all analyses. For descriptive analyses, we calculated absolute frequencies and percentages for discrete variables or means and standard deviations for continuous variables for participants without complications versus with at least one complication, respectively. Joint analyses of different exposure variables included all participants with information on the respective exposure variables (complete case analysis).

For each outcome variable, the crude overall risk was estimated as the percentage of patients with the outcome. Furthermore, we conducted log-binomial regression analyses with sex and diabetes duration as independent variables for all outcome variables [22]. In addition, we conducted univariable log-binomial regression analysis (Model 1) and multivariable log-binomial regression analysis adjusting for sex and diabetes duration (Model 2) for each exposure and the composite endpoint as the dependent variable. Continuous independent variables were modelled with natural cubic splines with three equally spaced knots in order to allow nonlinear associations [23].

To illustrate dose-response relationships, we plotted the model-based predicted risk against continuous exposures for female and male patients. Using the sex-adjusted model for diabetes duration, we estimated the risk with 95% confidence intervals (CI) of the composite endpoint after a 15-year diabetes duration for males and females separately. For categorical exposures, results are presented as relative risks (RRs) and 95% CI for each category. For continuous exposures, model-based RRs are reported for the mean and the midpoint of the upper quartile, with the midpoint of the lowest quartile as reference.

## 3. Results

In total, 4413 (survey_2009/10_: 2231; survey_2012/13_: 1009; survey_2015/16_: 1173) eligible persons received the questionnaires. 1875 (42%) (survey_2009/10_: 839 [38%]; survey_2012/13_: 452 [45%]; survey_2015/16_: 584 [50%]) of these took part in the survey. Information on the composite endpoint was available for 1789 (95%) (survey_2009/10_: 794 [95%]; survey_2012/13_: 434 [96%]; survey_2015/16_: 561 [96%]) patients. These latter patients were included in the analyses.  Table 1 shows characteristics of patients with and without complications/comorbidities. The mean diabetes duration and age were 12.4 years (range: 9.9–17.7 years) and 15.4 years (range: 11.3–21.9 years), respectively.

**Figure 1:**
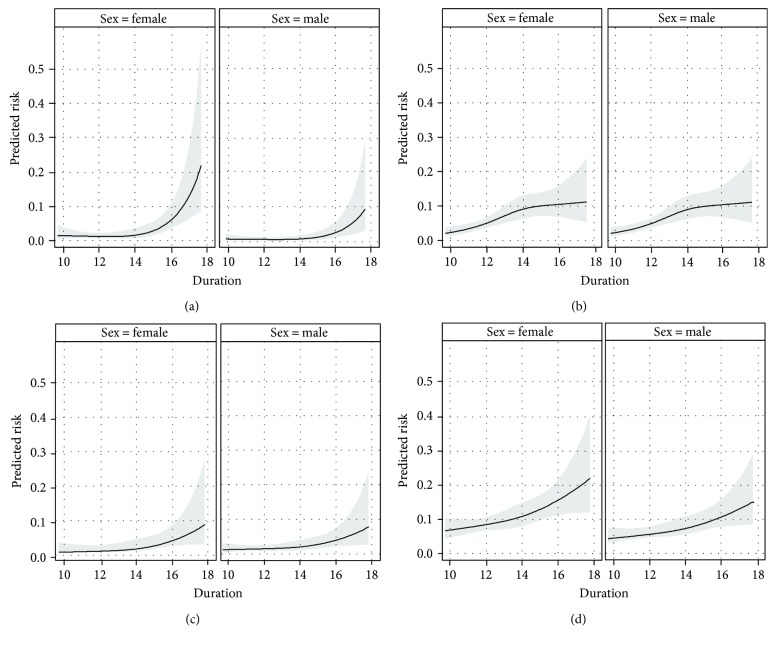
Risk of diabetes-related complications in relation to diabetes duration. Risk of retinopathy ((a) *N* = 1773), hypertension ((b) *N* = 1780), nephropathy ((c) *N* = 1775), and dyslipidemia ((d) *N* = 1772) with 95% confidence bands (shaded areas). The estimated risk was derived from log-binomial regression analyses with the respective outcome as the dependent variable and diabetes duration and sex as independent variables. Diabetes duration was modelled as a natural cubic spline with three equally spaced knots. Different *N* are due to missing values in the outcome variables.

The crude overall risks for DR, DN, hypertension, dyslipidemia, and the composite endpoint were 1.4% (95% CI: 0.8%–1.9%), 2.0% (95% CI: 1.3%–2.6%), 5.4% (95% CI: 4.3%–6.4%), 7.8% (95% CI: 6.6%–9.1%), and 14.1% (95% CI: 12.5%–15.7%), respectively.  Figure 1 illustrates the risks for the single outcomes dependent on diabetes duration estimated with spline regression. Except for hypertension, girls/women are estimated to have a higher risk than boys/men. The risk of DR and DN is close to zero in patients with a diabetes duration up to 14 years in cross-sectional analysis. Thereafter, the risk increases, particularly for DR. In contrast, the risks of hypertension and dyslipidemia already show an upward trend from year ten after diabetes duration onwards. The slope for dyslipidemia risk increases slightly with diabetes duration and shows the highest risks of all outcomes considered. The slope for hypertension shows a rather linear trend until year 14 since diagnosis and flattens thereafter.

**Figure 2:**
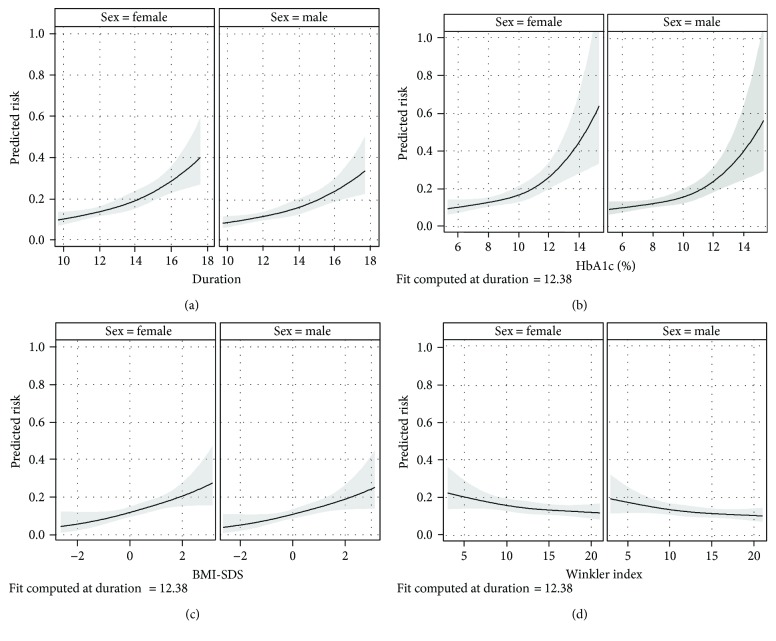
Risk of at least one diabetes-related complication in relation to different exposures. Risk of retinopathy, nephropathy, hypertension, or dyslipidemia (event = yes) in relation to diabetes duration ((a) *N* = 1789), HbA1c ((b) *N* = 1711), body mass index standard deviation scores ((c) *N* = 1741), and the socioeconomic status index (Winkler index) ((d) *N* = 1761) with 95% confidence bands at 12.4-year diabetes duration. The risk was estimated from separate log-binomial regression models for each exposure with the composite endpoint as the binary dependent variable and diabetes duration, sex, and the respective exposure as independent variables. Continuous variables were included as natural cubic splines with three equally spaced knots. Different *N* are due to missing values in the exposure variables.

Log-binomial regression for the composite endpoint with diabetes duration and sex as independent variables estimated the 15-year risk after diabetes diagnosis for female patients at 22.9% (95% CI: 18.8%–27.9%) and male patients at 19.2% (95% CI: 15.5%–23.8%). Associations between the risk and exposure variables are shown as RRs (Table 2) and dose-response relationships (Figure 2). We assessed a time trend by comparing risks between surveys. After adjustment for sex and diabetes duration, the risk in 2012/13 and 2015/16 was 22% and 25% lower, respectively, than that in 2009/2010.  Figure 2(a) suggests a slightly curved relationship between diabetes duration and the risk of at least one complication. Correspondingly, diabetes duration was associated with an increased risk independent of sex (Table 2).

Not living with the biological parents showed a tendency for an increased risk. After adjustment for diabetes duration and sex, the risk for the group with the highest SES (4th quartile) was reduced compared to the 1st quartile (Table 2).  Figure 2(d) indicates a continuously decreasing risk with increasing SES in an almost linear fashion.

BMI and PA were both associated with an increased risk (Table 2). Having a BMI in the 4th quartile versus the 1st quartile was associated with more than doubled risk. Furthermore, Figure 2(c) indicates an almost linear relationship between BMI and the risk of the composite endpoint.

A similar association was also seen for the 4th quartile of HbA1c compared to the 1st quartile (Table 2). However, this dose-response relationship (Figure 2(b)) is characterized by an exponential curve with a strongly increasing risk in higher HbA1c ranges. The RRs for SMBG and OII show no clear trends. Compared to more than 8 SMBG per day, the three categories with fewer SMBG were associated with a decreased risk in model 2. Between these lower categories, the risk decreased with increasing frequency of SMBG. Compared to patients never omitting insulin injections, omitting insulin injection more than once a day elevated the risk 1.91-fold (Table 2).

Potentially beneficial health care-related factors (CSII, DMP, and DHC) showed no clear association with the risk of the composite endpoint. However, participants who reported that they did not know whether they used the DHC showed a tendency to a lower risk compared to DHC users (Table 2).

## 4. Discussion

In our analysis comprising three cross-sectional surveys of early-onset and long-duration type 1 diabetes patients, we estimated the risk of DR, DN, hypertension, or dyslipidemia at about 19% (men) and 23% (women) after 15 years of diabetes duration. Analyses of temporal trends suggested a decreasing risk of the composite endpoint over the seven-year observation period, probably reflecting intensification and improvements in diabetes care. Indicators for glycemic control and SES as well as lifestyle-related factors such as BMI and PA were strongly associated with the risk for developing at least one of the complications.

Regarding the development of DR in Germany, Hammes et al. [5] found that the risk of DR starts to increase about 10 to 15 years after the onset of type 1 diabetes and being close to zero before. This is in line with our findings. However, the crude prevalence of any DR of 27.4% is hardly comparable since the mean age and diabetes duration (31.1 and 14.5 years, resp.) were higher than in our study. The same holds for the comparison with the results from Raile et al. [8], who investigated the risk of DN in a German cohort. Their survival curves with diabetes duration as a time scale correspond to our results in Figure 1 whereas the crude risk estimates are higher, probably due to higher mean age and diabetes duration. A joint analysis of the Diabetes Control and Complications Trial and the Pittsburgh Epidemiology of Diabetes Complications Study estimates the risk of DR and DN under modern-day treatment and after a 30-year diabetes duration at approx. 50% and 20%, respectively [24]. For DR, similar risk estimates are reported from Swedish registries for young adults between 18 and 41 years of age whereas the risk for DN is estimated at approximately 10% [25]. The results show a risk increase already before 10 years since diagnosis which may suggest differences in the clinical course between countries.

With regard to dyslipidemia, our risk estimates are considerably lower than in previous studies even in comparable age groups [10, 26]. To a lower extent, also higher prevalence of hypertension were reported (e.g., Schwab et al. [10]). In these studies, the outcome assessment was based on clinical measurements. In contrast, we relied on self-reports which might have led to underreporting of dyslipidemia and hypertension due to recall bias. Diagnosis of DN and DR might be less prone to recall bias, since patients may perceive DN and DR as more severe complications than dyslipidemia and hypertension. Furthermore, physicians may not always give a diagnosis to patients, despite elevated blood pressure and/or blood lipids.

Despite these differences, our findings on glycemic control as an important predictor for dyslipidemia [26], DR, DN [27], and hypertension [28] are in line with previous studies. Similarly, PA [29], BMI [30], and diabetes duration [5, 8] had been reported to associate with the risk of complications. With regard to the family and socioeconomic background, we found that not living with both biological parents increased the risk slightly. This is consistent with other studies that identified an association between family structure and glycemic control [12]. In addition, associations between SES [31] have been documented previously. Despite evidence for the use of CSII to improve glycemic control, we did not observe a protective association regarding diabetic complications, whilst others did (e.g., [32]). This could be explained, for instance, by the fact that we did not know for how long participants used CSII and if CSII was introduced before or after the onset of the complication(s). We found no protective association for patients taking part in a DMP. A systematic review of type 1 diabetes and type 2 diabetes DMPs concludes that DMPs can improve glycemic control modestly [33]. However, to our knowledge, there is no previous study which investigated the association between DMP and DHC in connection with complications in type 1 diabetes in Germany. With respect to DMPs on type 2 diabetes mellitus, some evaluation studies are in favor of DMP regarding survival [34, 35], whilst one study concludes that there are no differences between DMP participants and nonparticipants with regard to complications [36].

For the German general population, it is well known that SES is associated with many health outcomes leading to reduced life expectancy and increased mortality for groups with low SES (e.g., [37]). Less is known about health inequalities in the context of complications among type 1 diabetes patients. In the present study, we identified an inverse relationship between SES and complications. The differences we found might be caused, on the one hand, by diabetes-specific differences (e.g., glycemic control) and, on the other hand, by differences that also apply to the general population. For instance, it is known that BMI is inversely associated with SES among German children and adolescents [38], and we found BMI to be strongly associated with the risk of complications. One study found an inverse association between household income and the frequency of intentionally omitted insulin injections among adults but also reported high education to increase the risk of complications [39]. Future studies should investigate which pathways lead to social inequalities in the risk of complications in order to identify vulnerable groups and develop targeted interventions.

The central role of glycemic control to prevent complications is well acknowledged in clinical guidelines [40]. We used HbA1c as an indicator for long-term blood glucose levels and found a strong association with the risk of complications in an early-onset type 1 diabetes cohort. The exponential relationship between HbA1c and the risk of complications suggests that a one-unit increase in higher HbA1c ranges increases the risk more strongly than that in lower ranges. Thus, there might be a disproportionate potential for risk reduction in patients with high HbA1c values. In order to reduce complications in early-onset type 1 diabetes, it is essential to investigate underlying causes of elevated blood glucose.

Our results imply that participation in DMP and the use of the DHC were not associated with a reduced risk of complications. These programs are, amongst other aims, designed to prevent complications; wherefore, one would expect a reduced risk. Our negative results might call for improvement of these programs with special focus on the early-onset type 1 diabetes population. However, before drawing final conclusions on the effectiveness of DMP and use of a DHC, further evaluation is needed since we did not account for variables that potentially influence participation and also related to the risk of complications.

### 4.1. Strengths and Weaknesses

Our results are based on self-reports which may have led to measurement error and misclassification. Especially for nephropathy and retinopathy, direct standardized measurement would have been preferable to distinguish, for example, retinopathy with and without visual impairment. However, the fact that our dose-response curve for DN (Figure 1) corresponds to the survival curve for DN from Raile et al. [8] might suggest reasonably valid self-reported data on complications. Furthermore, by asking explicitly for physician-diagnosed retinopathy and nephropathy, we tried to improve the validity of the self-reported data, since most T1D patients in Germany are treated in diabetes centers under the same guidelines. In addition, under the assumption that the degree of the outcome misclassification does not depend on the values of the respective exposures, the shape of the dose-response curves can still be considered valid. With regard to HbA1c, a previous analysis using the data of survey_2009/10_ showed good accordance of self-reported HbA1c with clinical documentation [16]. Nevertheless, we were not able to account for different laboratory methods of HbA1c, which recently have been suggested to influence HbA1c results [41].

Unfortunately, we had no information on puberty status, which has been suggested to be an important factor for the risk of complications [42, 43]. Our study might be prone to selection bias, since additional analyses showed that nonresponders were younger and had a longer diabetes duration. Furthermore, nonresponders who sent back a short questionnaire reported poorer overall health compared to participants with full questionnaires (results not shown). A major limitation is the fact that we collected exposure information retrospectively; wherefore, we could not establish a time order between exposure and outcome. Thus, all results should be considered exploratory. With regard to the statistical analyses, we defined a composite outcome variable to increase the number of events and thus the statistical power. However, it is probable that the relationships between exposure and the respective outcomes differ, which cannot be assessed with our results.

One strength of our study is the fact that it is built on a nationwide sample, drawn from a register with 97% coverage of all early-onset type 1 diabetes cases in Germany [15]. Furthermore, we were able to include sociodemographic and health care-related variables, which are often not available in clinical registries. In addition, we determined a time trend of the risk of complications between 2009/10 and 2015/16. With regard to the analyses, we used natural cubic spline regressions, which has the advantage of maintaining the continuous nature of exposure variables instead of artificially categorizing it in case of a nonlinear relationship. Therefore, we kept information, allowing us to characterize dose-response relationships. The use of log-binomial regression has the advantage of directly estimating RRs instead of odds ratios as is the case in logistic regression. Thus, we could avoid overestimating of RRs when the rare disease assumption does not hold.

### 4.2. Conclusion

Altogether, we provide evidence that in early-onset type 1 diabetes, there exists a considerable risk of complications and their predictors already in young ages. However, we observed a decreasing risk over time probably representing improved care among younger birth cohorts. Differences in the risk of complications with regard to SES, lifestyle-related variables, and glycemic control call for further improvement of care and development/implementation of prevention programs. Future research on early-onset type 1 diabetes should focus on prevention of diabetic complications in young ages and clarification of pathways of the associations found in this study.

## Figures and Tables

**Table 1:** Characteristics of the study population with type 1 diabetes onset in preschool age and diabetes duration of at least 10 years.

Variable (*n* missing)	Total cohort	No complication^∗^	At least one complication^†^
*N*	1.789	1.537	252
Survey wave (0)			
2009/10	794 (44.4)	649 (42.2)	145 (57.5)
2012/13	434 (26.3)	386 (25.1)	48 (19.1)
2015/16	561 (31.4)	502 (32.7)	59 (23.4)
Female sex (0)	874 (48.9)	739 (48.1)	135 (53.6)
Age in years (0)	15.4 ± 2.0	15.2 ± 2.0	16.2 ± 2.2
Age at onset (0)	3.0 ± 1.2	2.9 ± 1.2	3.2 ± 1.1
Diabetes duration in years (0)	12.4 ± 1.7	12.3 ± 1.6	13.0 ± 2.0
Hypertension (9)	96 (5.4)	—	96 (39.51)
Dyslipidemia (17)	139 (7.8)	—	139 (59.15)
Retinopathy (16)	24 (1.4)	—	24 (10.17)
Nephropathy (14)	35 (2.0)	—	35 (14.71)
Socioeconomic status index^‡^ (28)	13.4 ± 4.4	13.5 ± 4.4	12.6 ± 4.4
Living with… (6)			
Both biological parents	1.382 (77.5)	1.198 (78.2)	184 (73.3)
Else	401 (22.5)	334 (21.8)	67 (26.7)
BMI-SDS (48)	0.30 ± 0.90	0.26 ± 0.89	0.54 ± 0.89
Freq. of vigorous physical activity (24)			
Never	113 (6.4)	86 (5.7)	27 (10.8)
1-2 times/month	155 (8.8)	126 (8.3)	29 (11.7)
1-2 times/week	717 (40.6)	609 (40.2)	108 (43.4)
More than 1-2 times/week	780 (44.2)	695 (45.8)	85 (34.1)
HbA1c in % (78)	8.2 ± 1.3	8.2 ± 1.3	8.6 ± 1.7
HbA1c in mmol/mol (78)	66 ± 15	66 ± 14	70 ± 18
Freq. of SMBG (35)			
0–2/day	81 (4.6)	65 (4.3)	16 (6.5)
3–5/day	853 (48.6)	718 (47.7)	135 (54.4)
6–8/day	676 (38.5)	602 (40.0)	74 (29.8)
>8/day	144 (8.2)	121 (8.0)	23 (9.3)
Freq. of omitted insulin injections (40)			
Never	520 (29.7)	450 (30.0)	70 (28.2)
1-2 times/week	789 (45.1)	675 (45.0)	114 (46.0)
3–5 times/week	309 (17.7)	268 (17.9)	41 (16.5)
(almost) 1 time/day	103 (5.9)	87 (5.8)	16 (6.5)
More than 1 time/day	28 (1.6)	21 (1.4)	7 (2.8)
Insulin therapy (18)			
1–3 injections/day	111 (6.3)	91 (6.0)	20 (8.0)
≥4 injections/day	647 (36.5)	565 (37.2)	82 (32.7)
CSII	1.013 (57.2)	864 (56.8)	149 (59.4)
Participation in DMP (19)			
No	767 (43.3)	666 (43.8)	101 (40.4)
Yes	686 (38.8)	583 (38.4)	103 (41.2)
Not known	317 (17.9)	271 (17.8)	46 (18.4)
Use of diabetes health card (17)			
No	911 (51.4)	787 (51.7)	124 (49.8)
Yes	777 (43.9)	660 (43.3)	117(47.0)
Not known	84 (4.7)	76 (5.0)	8 (3.2)

Data are *N* (%) or mean ± SD. Abbreviations: BMI-SDS: body mass index standard deviation score; SMBG: self-monitoring of blood glucose; CSII: continuous subcutaneous insulin injection; DMP: disease management program; ^∗^persons who reported to have never been diagnosed with hypertension, dyslipidemia, retinopathy, or nephropathy; ^†^persons who reported to have ever been diagnosed with at least one of hypertension, dyslipidemia, retinopathy, or nephropathy; ^‡^range 3–21—higher values indicate higher socioeconomic status.

**Table 2:** Relative risks (RRs) with 95% confidence intervals (95% CI) for the risk of have ever been diagnosed with hypertension, dyslipidemia, retinopathy or nephropathy.

Exposure^∗^	RR from model 1 (95% CI)	RR from model 2 (95% CI)
Sex (*N* = 1.789)		
Male	1.00	1.00
Female	1.21 (0.96–1.52)	1.19 (0.95–1.49)
Survey wave (*N* = 1.789)		
2009/10	1.00	1.00
2012/13	0.61 (0.45–0.82)	0.78 (0.56–1.09)
2015/16	0.58 (0.43–0.76)	0.75 (0.54–1.03)
Diabetes duration^†^ (*N* = 1.789)		
10.5 years	1.00	1.00
12.4 years	1.34 (1.04–1.73)	1.34 (1.03–1.72)
15.4 years	2.32 (1.67–3.23)	2.31 (1.66–3.21)
Socioeconomic status index^†‡^ (*N* = 1.761)		
6.5	1.00	1.00
13.0	0.67 (0.48–0.92)	0.75 (0.55–1.04)
19.0	0.57 (0.41–0.79)	0.67 (0.48–0.92)
Living with… (*N* = 1.783)		
Biological parents	1.00	1.00
Else	1.25 (0.97–1.62)	1.16 (0.90–1.50)
BMI-SDS^†^ (*N* = 1.741)		
−1.5	1.00	1.00
0.3	1.75 (1.07–2.88)	1.72 (1.05–2.82)
2.0	2.89 (1.77–4.72)	2.64 (1.61–4.33)
Freq. of physical activity (*N* = 1.765)		
Never	2.19 (1.49–3.22)	1.76 (1.19–2.61)
1-2 times/month	1.72 (1.17–2.52)	1.45 (0.99–2.14)
1-2 times/week	1.38 (1.06–1.80)	1.30 (0.99–1.70)
More than 1-2 times/week	1.00	1.00
HbA1c^†^ (*N* = 1.711)		
6.3% (45 mmol/mol)	1.00	1.00
8.2% (66 mmol/mol)	1.28 (1.01–1.63)	1.23 (0.97–1.57)
12.1% (109 mmol/mol)	2.67 (1.78–4.01)	2.52 (1.70–3.72)
Freq. of SMBG (*N* = 1.706)		
0–2/day	1.24 (0.69–2.20)	0.88 (0.49–1.59)
3–5/day	0.99 (0.66–1.49)	0.80 (0.53–1.21)
6–8/day	0.69 (0.45–1.06)	0.64 (0.41–0.99)
>8/day	1.00	1.00
Freq. of omitted insulin injections (*N* = 1.749)		
Never	1.00	1.00
1-2 times/week	1.07 (0.81–1.41)	1.07 (0.81–1.40)
3–5 times/week	0.99 (0.69–1.41)	0.98 (0.68–1.39)
(almost) 1 time/day	1.15 (0.70–1.90)	1.08 (0.66–1.77)
More than 1 time/day	1.86 (0.94–3.66)	1.91 (1.03–3.53)
Insulin therapy (*N* = 1.771)		
1–3 injections/day	1.23 (0.80–1.87)	1.15 (0.76–1.74)
4+ injections/day	0.86 (0.67–1.11)	0.80 (0.62–1.03)
CSII	1.00	1.00
Participation in DMP (*N* = 1.770)		
Yes	1.00	1.00
No	0.88 (0.68–1.13)	0.91 (0.71–1.17)
Do not know	0.97 (0.70–1.33)	0.95 (0.69–1.30)
Use of diabetes health card (*N* = 1.772)		
Yes	1.00	1.00
No	0.90 (0.72–1.14)	0.92 (0.73–1.15)
Do not know	0.63 (0.32–1.25)	0.55 (0.28–1.08)

Unadjusted RRs were derived from separate log-binomial regression with the composite outcome as the dependent binary variable and the respective exposure variables as the independent variable (model 1). Model 2 adjusted for sex and diabetes duration. Abbreviations: BMI-SDS: body mass index standard deviation scores; SMBG: self-monitoring of blood glucose; CSII: continuous subcutaneous insulin injection; DMP: disease management program; ^∗^different *N* due to missing values in exposure variables; ^†^modelled as a natural cubic spline with three equally spaced knots—estimates are model-based RR for the mean and midpoint of the fourth quartile versus the midpoint of the first quartile; ^‡^range 3–21—higher values indicate higher socioeconomic position.
